# The role of ecdysis in repair of an attachment system: a case study using geckos

**DOI:** 10.1242/jeb.245286

**Published:** 2023-05-12

**Authors:** Rishab R. Pillai, Jendrian Riedel, Lin Schwarzkopf

**Affiliations:** ^1^ College of Science and Engineering, James Cook University, Townsville 4814, Australia; ^2^ Evolutionary Biology, Bielefeld University, Bielefeld 33615, Germany; ^3^ Herpetology Section, Zoological Research Museum Alexander Koenig (ZFMK) - Leibniz Institute for the Analysis of Biodiversity Change (LIB), Bonn 53113, Germany

**Keywords:** Attachment, Damage, Diplodactylidae, Ecdysis, Integument, Geckos, Safety factors

## Abstract

Skin provides functions such as protection and prevention of water loss. In some taxa, the outer surface of skin has been modified to form structures that enable attachment to various surfaces. Constant interaction with surfaces is likely to cause damage to these attachment systems and reduce function. It seems logical that when skin is shed via ecdysis, its effectiveness will increase, through repair of damage or other rejuvenating mechanisms. We address two questions using three diplodactylid geckos as model species. (1) Does repeated mechanical damage affect clinging ability in geckos to the point that they cannot support their own body weight? (2) Does use without induced damage reduce effectiveness of the attachment system, and if so, does ecdysis restore clinging ability? We found that repeated damage reduced clinging ability in all three species, although at different rates. Additionally, use reduced clinging ability over time when no apparent damage was incurred. Clinging ability increased after ecdysis in all three species, both when damage was specially induced, and when it was not. After normal use without induced damage, the increase in clinging ability after ecdysis was statistically significant in two of three species. Our findings show that use decreases clinging ability, and mechanical damage also effects geckos' capacity to exert shear forces consistently. Thus, ecdysis improves clinging ability both in scenarios where damage is induced and more generally. In addition to the physiological functions provided by skin, our study highlights an important function of ecdysis in a speciose vertebrate group.

## Teaser

**Highlighted Article:** Ecdysis restores clinging ability in geckos reduced by normal use and damage.

## INTRODUCTION

Effective movement within the environment is essential for prey capture, predator avoidance and mate acquisition (Alexander, 2003; Betz and Kölsch, 2004). Movement in taxa such as spiders (*Aphonopelma seemanni* and *Cupiennius salei*; Niederegger and Gorb, 2006), dock beetles (*Gastrophysa viridula*; Bullock and Federle, 2011), stick insects (*Carausius morosus*; Labonte et al., 2019), cockroaches (*Nauphoeta cinerea*; Clemente et al., 2009) and geckos (*Gekko gecko* and *Hemidactylus garnoti*, Autumn and Peattie, 2002) is dependent upon their attachment mechanisms, which are associated with modifications of the outer epidermal generation (OEG). These structures differ morphologically among taxa and the mechanisms of attachment vary. Despite the morphological differences, the function of these attachment systems is dependent on interactions with substrates upon which movement occurs (Pillai et al., 2020a,b; Burack et al., 2022). During these interactions potential damage and contamination might occur, reducing the effectiveness and function. Processes such as self-cleaning could potentially reduce the effect of contamination (Clemente et al., 2010; Hansen and Autumn, 2005); however, the process of ‘ecdysis’ or ‘skin-shedding’ repairs damage and wounds that could potentially restore function (Lai-Fook, 1968; Weis, 1976; Vafopoulou, 2009; Pellett and O'Brien, 2019).

The removal and replacement of the OEG is a trait common across terrestrial vertebrates (Maderson, 1966): in a process known as ‘shedding’, ‘sloughing’ or ‘ecdysis’, the old OEG is removed after a new one is formed from the underlying living tissue. Some clades shed their OEG constantly, for example, the mammalian epidermis is produced and replaced continuously through movement of new cells into the cornified layers (Maderson, 1965; 1967). In contrast, squamate reptiles shed periodically, losing the entire outer epidermal generation at once. The process of shedding may have evolved to keep pace with somatic growth and to rid the surface of parasites and other pollutants (Böhme and Fischer, 2000). Shedding also likely repairs external damage caused by the environment (Fushida et al., 2020).

Squamates have a multilayered and complex epidermis: the stratum germinativum is the deepest epidermal layer, preceding its derivatives, the inner epidermal generation (IEG) and the OEG, which consist of three layers each (Maderson, 1964). The outermost part of the top layer of the OEG, termed Oberhäutchen, gives rise to microscopic derivates called microornamentation or, more precisely, epidermal outgrowths (Irish et al., 1988; Maderson et al., 1998; Garner and Russell, 2021). One example of epidermal outgrowths are the hairlike structures, termed ‘setae’ (Garner and Russell, 2021), which are responsible for adhesion of the toepads of geckos (family Gekkota; Ernst and Ruibal, 1966), some iguanians (Anolidae and Polychrotidae, Ruibal and Ernst, 1965) and few species of scincids (Williams and Peterson, 1982). The adhesive pads consist of modified scale rows, lamellae and scansors, bearing millions of setae, which are critical for effective locomotion, and have enabled these groups to adhere to vertical and inverted substrates (Russell and Johnson, 2007, 2014; Gamble et al., 2012). Setae enable attachment through a combination of van der Waals forces (Autumn et al., 2000; Autumn and Peattie, 2002), capillary interactions (Huber et al., 2005; Puthoff et al., 2010; Mitchell et al., 2020), acid–base interactions (Singla et al., 2021) and possibly, electrostatic interactions (Izadi et al., 2014).

Early studies of gecko adhesion found that adhesive capabilities decreased with increased time since shedding. These studies suggested that clumping of setae caused by fouling, rather than damage, may have caused the decrease (Dellit, 1934; Hiller, 1968). On the other hand, a decline in adhesion over time since shedding is difficult to reconcile with more recent work suggesting that, on a micro-scale, individual seta are resistant to, or undergo little wear with repeated use (Gravish et al., 2010; Autumn et al., 2014), apparently suffering no reduction in adhesion and friction. It is possible that fouling and clumping, or damage to entire lamellae or pads at the macro-scale, rather than of individual setae, caused the reduced performance observed in early studies (Dellit, 1934; Hiller, 1968). If so, it is likely that shedding and consequent rejuvenation of the adhesive system, restores attachment capabilities.

Several studies have investigated the role of ecdysis in wound repair (Lai-Fook, 1968; Weis, 1976; Vafopoulou, 2009; Pellett and O'Brian, 2019); however, the role of ecdysis in repairing damage and rejuvenating the function of attachment systems has rarely been investigated. Hiller (1968) suggested that ecdysis might restore the attachment system of geckos after exposure to contamination and damage. Attachment of geckos is the culmination of contact on the macro (lamellae and scansors), micro (setal fields) and nano (spatulae) levels that could be exposed to damage through use and interaction with substrates. Therefore, using geckos as a model system, we examine how ecdysis affects performance when the attachment system is used normally, and when damage is induced. Specifically, we: (i) quantified the effect of mechanical damage on clinging ability of pad-bearing diplodactylid geckos, including quantifying safety factors for clinging, and (ii) tested whether ecdysis improves clinging ability when the attachment system is used for a period of time between shedding events. We hypothesised that both damage and general use would reduce clinging ability, and that ecdysis would repair and rejuvenate setal fields, leading to an improvement in clinging in both scenarios.

## MATERIALS AND METHODS

### Study species and husbandry

We examined clinging performance in three scansorial diplodactylid gecko species, *Oedura castelnaui* (Thominot 1889) (*N*=10 in experiment 1 and *N*=9, in experiment 2, 4 males and 6 females), *Oedura monilis* De Vis 1888 (*N*=11, 5 males and 6 females) and *Strophurus krisalys* Sadlier et al. 2005 (*N*=6, 2 males and 4 females), all of which have leaf-like terminal toepads but different toepad areas relative to body size (Pillai et al., 2020a,b). Geckos were housed at James Cook University, Townsville, Australia, in constant temperature rooms with a 12 h:12 h light:dark inverted photoperiod. Each gecko was housed individually in a plastic enclosure lined with paper towel, containing a ceramic tile hide, and provided with water *ad libitum*. Geckos were fed four large domestic crickets (*Acheta domestica*) once a week. All enclosures were placed on heat mats at 32°C, running along one end of the enclosure, to provide a thermal gradient. Information on the geographic origin of the animals and capture methods are provided elsewhere (Pillai et al., 2020a; Riedel et al., 2020).

Fieldwork to collect and house animals was undertaken under permits ‘To Take, Use, Keep or Interfere with Cultural or Natural Resources (Scientific purposes)’, Nature Conservation (administration) Regulation 2006 WITK18258517 and Scientific Purposes Permit ‘Taking a protected animal for scientific purposes’ WA0005590. Clinging ability experiments were undertaken under James Cook University Ethics Permit A2691.

### Measuring toe pad area

Surface area of the adhesive toepads may influence clinging performance (Irschick et al., 1996; Russell and Johnson, 2007, 2014; Peattie, 2009); therefore, surface area of adhesive regions was measured prior to testing, and total surface area, not excluding damaged areas, was included in all statistical analysis. The palmar and plantar surfaces of all individuals of the three species were photographed through glass against a uniform dark background with a scale in each image. To adjust the contrast and highlight the adhesive regions (both lamellae and scansors), we used Lightroom CC (Adobe). We used the incorporated scale bar to calibrate measurements in ImageJ (Schneider et al., 2012; Gillies et al., 2014). The ‘thresholding’ feature was then used to select the toepads by manipulating the saturation of the photo, as they contrasted strongly with the rest of the image. Measurements were taken for all five toes on the right hand (manus) and right foot (pes) of all geckos, and doubled to calculate total adhesive area for each gecko.

### Shedding intervals

From 23 March 2020 to 31 December 2020, we monitored geckos daily for shedding. Geckos were marked using a non-toxic Sharpie™ pen, which does not affect the rate of skin shedding in these gecko species (Fushida et al., 2020). When a shedding event was detected after the mark disappeared, the date, temperature and humidity of the enclosure were recorded. Shedding intervals (number of days) were variable within and among species, and are provided in Fig. S1.

### Damage and ecdysis (experiment 1)

Five days after a shedding event, we tested each gecko three times to quantify maximum shear force generated on glass. Then, to cause accumulated damage, clinging ability was tested on glass again at ten-day intervals until the next shedding event. All three species were tested four times within a shedding cycle. We artificially induced damage to the entire system comprising of both scansors and lamellae as we could not distinguish scansors from lamellae with certainty owing to lack of information about the internal morphology of diplodactylid gecko toes (Russell and Bauer, 1988; Russell et al., 2019).

We recorded the maximum force generated by the whole gecko (four limbs) using a force gauge (Extech 475040, Extech Equipment Pty Ltd) attached to a fishing line (diameter 0.5 mm) looped around the inguinal region of the gecko (Niewiarowski et al., 2012). Each individual was tested three times to constitute one trial. To ensure that the attachment system of the gecko was engaged completely, each gecko was allowed to take one step with each of the four limbs. Once contact was made with all four limbs, geckos were pulled backwards horizontally at an angle of zero degrees and a constant velocity of ∼0.5 cm s^−1^ (a ruler was placed beside the gecko and used in conjunction with a stopwatch). The test surface was cleaned using Kimwipes™ and reverse osmosis water, and air-dried for 15 min between individuals. Only one investigator (R.R.P.) conducted clinging performance trials to reduce experimenter-specific effects (Pillai et al., 2020a,b).

We repeatedly tested shear force on glass to induce damage. Glass provides a greater attachment area for the setal fields than naturally rough surfaces and permits greater clinging ability due to the attachment system adhering firmly as a result of high levels of contact (Pillai et al., 2020b). This high level of adhesion can lead to damage, removing lamellae by partially rupturing, or entirely removing scansors or lamellae (Autumn et al., 2006). Visible damage to lamellae or scansors was recorded for each gecko.

### Use and ecdysis (experiment 2)

**Fig. 1.**
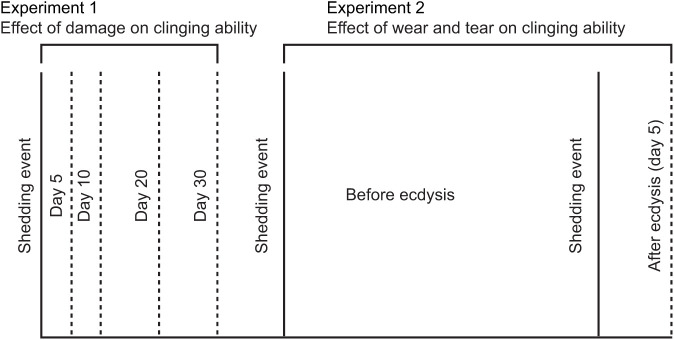
**Experimental timeline of the study.** Effect of mechanical damage (experiment 1) on clinging ability. To induce repeated damage, we tested animals at 5-day and then at 10-day intervals following shedding. To allow damage to the attachment apparatus to repair and rejuvenate, geckos were allowed to complete a shedding event prior to testing of the influence of time and ecdysis only on clinging ability (experiment 2). Geckos were tested once before (1–43 days) shedding and once after (5 days, one individual was tested 7 days after). *Oedura castlenaui* (*N*=10 in experiment 1, *N*=9 in experiment 2), *Oedura monilis* (*N*=11) and *Strophurus krisaly*s (*N*=6).

Following experiment 1, geckos underwent one shedding event before being used in experiment 2. This time, we quantified the role of ecdysis in renewing clinging ability, when no damage was inflicted. The setal fields were subjected only to normal substrate interactions on paper towel, the ceramic shelter or plastic container walls while walking or running within their enclosure. Thus, after we completed trials investigating the role of damage on shear force, geckos were rested until the following shedding event. Then, to determine if clinging ability was restored by shedding, we measured shear force generated prior to, but as close to shedding as possible. Because the timing of shedding is variable among individuals and species, we could not predict exactly when shedding would occur, and we did not want to test individuals repeatedly for this experiment, so we tested them close to when we thought they might shed (1–45 days before, Fig. S2). Then, each gecko was tested exactly 5 days after shedding (except one individual, which was tested 7 days after), to determine if shear force increased after shedding. Clinging ability was recorded using the same methods described above. As before, only one investigator (R.R.P.) conducted clinging performance trials to reduce experimenter-specific effects. A timeline of the tests we performed on shear force to investigate the effect of damage (experiment 1), and to investigate the effect of ecdysis following use (experiment 2) is shown in Fig. 1.

### Safety factors

We calculated safety factors under both scenarios, damage (experiment 1) and general use (experiment 2). Safety factors represent the margin of security-mitigating risks, in this case, the amount of force that can be applied in excess of that required to support an organism's body mass (Russell and Johnson, 2014) and reflect the capacity of a system to perform beyond expected or normal limits (Niewiarowski et al., 2016). For example, when all spatulae make contact with a substrate (under laboratory conditions), attachment forces are several times greater than what is needed to support a gecko's body weight (Autumn et al., 2000, 2006; Higham et al., 2017). This gave us an indication of safety factors under a scenario with substantial mechanical damage and also after normal use for locomotion. We calculated safety factors for experiment 1 and experiment 2, by dividing the shear force imparted by each gecko by its mass, to examine if the damage we inflicted affected attachment to the point where geckos could not support their own body weight. We recorded shear force in Newtons, which was converted to gram force mm^−1^ by multiplying shear force values by 101.97, which accounted for acceleration due to gravity (Russell and Johnson, 2014).

### Statistical analysis

All statistical analyses were conducted in R v. 4.0.3 (https://www.r-project.org/). Linear mixed effects models (LME models) were implemented with the package lme4 (https://cran.r-project.org/package=lme4; Bates et al., 2015), while model comparison was conducted using the package MuMin (https://cran.r-project.org/package=MuMIn). ANOVAs on all models were run using the package car (https://cran.r-project.org/package=car; Fox and Weisberg, 2019) and *post hoc* comparisons were implemented with the emmeans package (https://cran.r-project.org/package=emmeans). Toepad area was not significantly different between males and females; therefore, we pooled sexes for all statistical analyses (Fig. S3).

### Damage and clinging ability (experiment 1)

To quantify the effect of mechanical damage on clinging ability, we used log-transformed shear force per individual as the response variable in LME models as the distribution was right-skewed. Our initial set of models included ‘days since shedding’ and ‘species’ as individual fixed effects. These models included either ‘mass’ and ‘toepad area’ together, or just mass, or just toepad area. Each of these latter variables were included as either an offset or a scaling factor, as the units of measurements were in grams (mass) and mm^2^ (toepad area), producing six models. Additionally, two models included an interaction between ‘species’ and ‘days since shedding’, and both ‘mass’ and ‘toepad area’, either as offsets or scaled. As each gecko was tested multiple times in each trial at each interval, we added individual gecko IDs as random effects in each model to account for effects of repeated measures on individuals. This process gave us eight candidate models, which we compared with each other and the null model using the Akaike's information criterion (AIC; Table 1)*.* Models with the lowest AICc values were considered the best models explaining the effect of damage on shear force. The relative importance of the fixed terms included in the top models were determined using a type III ANOVA and *post hoc* comparisons were made using estimated least squares means*.* Geckos may not exhibit peak attachment capabilities immediately following shedding (Hiller, 1968); therefore, we used the maximum measure of attachment (rather than the first measure of attachment) to compare to the last (post-damage) measure of attachment to quantify the loss of clinging ability caused by damage.

**Table 1.**
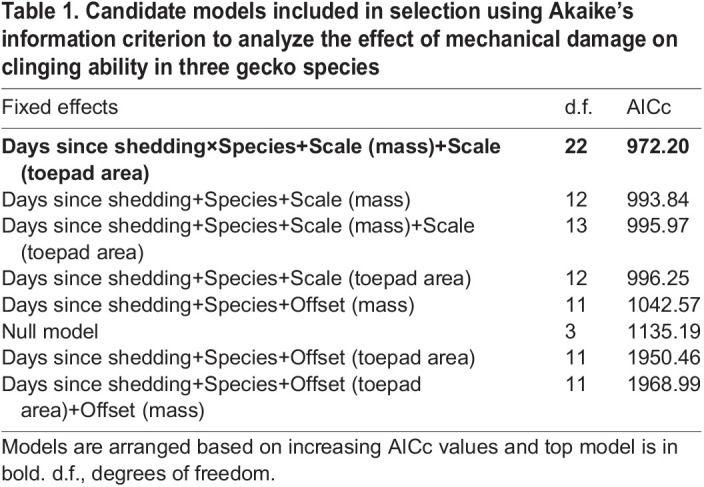
Candidate models included in selection using Akaike's information criterion to analyze the effect of mechanical damage on clinging ability in three gecko species

### General use and ecdysis (experiment 2)

To investigate how ecdysis influenced performance after use without inducing damage, we created four specific candidate linear mixed effects (LME) models to both investigate and correct for the effects of mass and toepad area. Natural-log-transformed measures of shear force per individual before and after the shedding event (experiment 2) were used as response variables in all candidate models to normalize a right-skewed distribution. Two models included the three-way interaction among ‘species’, ‘treatment’ (before or after) and ‘days before shedding’, while two models included these variables as fixed effects only, with no interactions. All models included mass and toepad area, either as scaled fixed effects or as offsets, in two models each (Table 2). The same gecko was tested three times before and three times after ecdysis, so individual gecko IDs were included as random effects in all candidate models. The models with the lowest AICc were considered the most likely to accurately predict the effect of ecdysis on shear force and were further analyzed using a type III ANOVA to understand the relative importance of fixed effects, following which, differences among variables were examined using estimated least squared means *post hoc* comparisons.

**Table 2.**
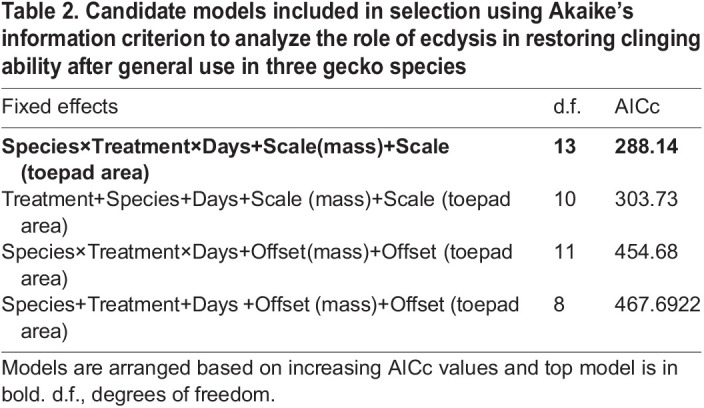
Candidate models included in selection using Akaike's information criterion to analyze the role ecdysis in restoring clinging ability after general use in three gecko species

### Clinging ability after damage and after general use

We used linear mixed-effects model to compare the impact of damage and general use on declines in shear force. Shear force was the response variable, with ‘species’ and ‘test period (shear force after damage and after general use) as fixed effects. We also included an interaction between ‘species’ and ‘test period’ in our model. As each individual was tested multiple times during each trial, individual gecko IDs were included as random effects in the model.

### Safety factors

Safety factors for each individual were calculated from experiment 1 and experiment 2. We conducted separate analyses to test the effect of damage (experiment 1) and general use (experiment 2). Each analysis consisted of four candidate LME models. To analyze the effect of damage, we used safety factors calculated from shear force exerted at four intervals (5, 10, 20 and 30 days). Models included the interaction between ‘days since shedding’ and ‘species’, and also ‘species’ and ‘days since shedding’ additively as fixed effects. Additionally, we tested models including only ‘species’ or only ‘days since shedding’ as fixed effects. The same set of fixed effects was used in candidate models to investigate safety factors before and after ecdysis, with the response variables being safety factors before and after shedding. In model sets of candidate models, response variables were log-transformed to normalize a right-skewed distribution. In both experiments, safety factor values were calculated for multiple measures per individual, so individual gecko IDs were included as random effects. The relative importance of the fixed terms included in the top model were determined using a type III ANOVA, and *post hoc* comparisons were made using estimated least squares means in the package emmeans (Table 3).

**Table 3.**
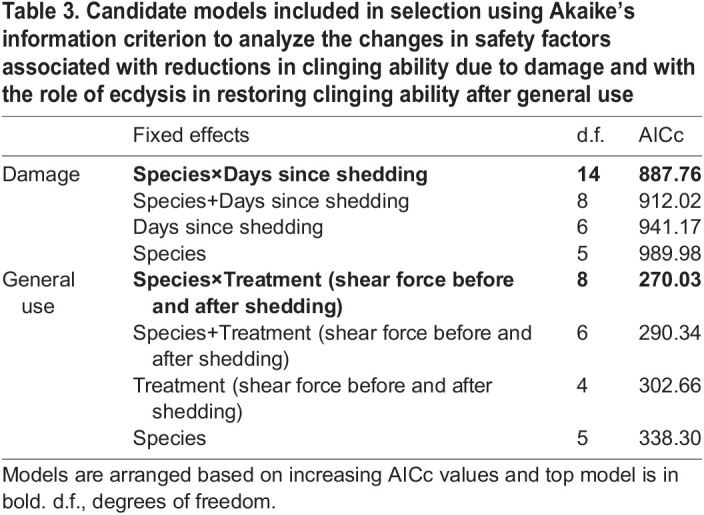
Candidate models included in selection using Akaike's information criterion to analyze the changes in safety factors associated with reductions in clinging ability due to damage and with the role of ecdysis in restoring clinging ability after general use

## RESULTS

### Damage and clinging ability (experiment 1)

The best model predicting the effect of damage on shear force included an interaction between ‘days since shedding’ and ‘species’, and ‘mass’ and ‘toepad area’ as scaled fixed effects (*R*^2^ conditional=0.77). Slopes of plots of clinging ability over time after repeated testing were negative, indicating that clinging ability always declined as damage increased. The slope of these relationships (or the rate at which clinging ability declined with damage) differed among species, indicated by the significant interaction between ‘days since shedding’ and ‘species’ (*P*<0.01, χ^2^=46.61, ANOVA, Type III Wald χ^2^ tests). Mass (*P*=0.21) and toepad area (*P*=0.63) had no significant effect on shear force (ANOVA, Type III Wald χ^2^ tests).

**Fig. 2.**
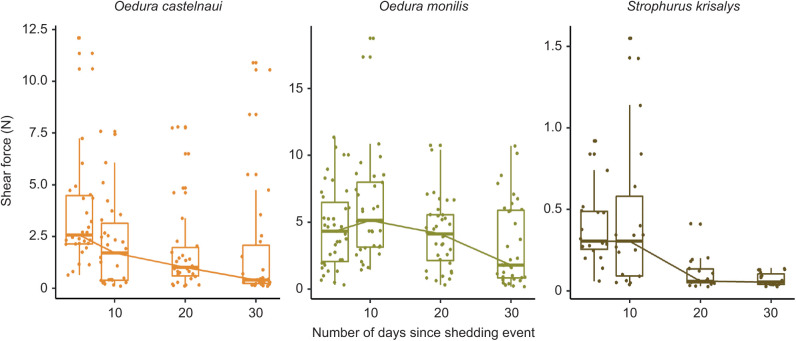
**The effect of mechanical damage on clinging ability of three gecko species.** Differences in absolute shear force (median±s.e.) with repeated damage. Following repeated damage, *O. castelnaui* exerted 1.90 N less shear force 30 days after shedding compared with 5 days after shedding (*N* =10, *P*<0.01, estimated marginal least square means *post hoc* comparisons); *O. monilis* exerted 2.70 N less shear force between day 10 and day 30 since shedding (*N*=11, *P*=0.05, estimated marginal least square means *post hoc* comparisons) and clinging ability decreased by 0.52 N between day 5 and day 30 in *S. krisalys* (*N*=6, *P*<0.01, estimated marginal least square means *post hoc* comparisons). Color palette using R package colRoz (https://jacintak.github.io/project/colRoz).

After damage, clinging ability decreased significantly in *O. castelnaui* (*N*=10, test day 5 versus 30, *P*<0.01, estimated marginal least square means *post hoc* comparisons) and *S. krisalys* (*N*=7, test day 5 versus 30, *P*<0.01, estimated marginal least square means *post hoc* comparisons). In *O. monilis*, the decrease in clinging ability was marginally significant (*N*=11, test day 5 versus 30, *P*=0.05, estimated marginal least square means *post hoc* comparisons). After ecdysis, clinging ability improved and all species exhibited peak clinging ability on the first day they were tested (5 days after shedding), except for *O. monilis*, which generated the highest clinging ability in the test 10 days after shedding. Clinging ability reduced by 1.90 N between day 5 and day 30 in *O. castelnaui* (*N*=10), by 2.70 N between day 10 and day 30 in *O. monilis* (*N*=11) and by 0.52 N between day 5 and day 30 in *S. krisalys* (*N*=7; Fig. 2).

### Macroscopic damage to toepads

**Fig. 3.**
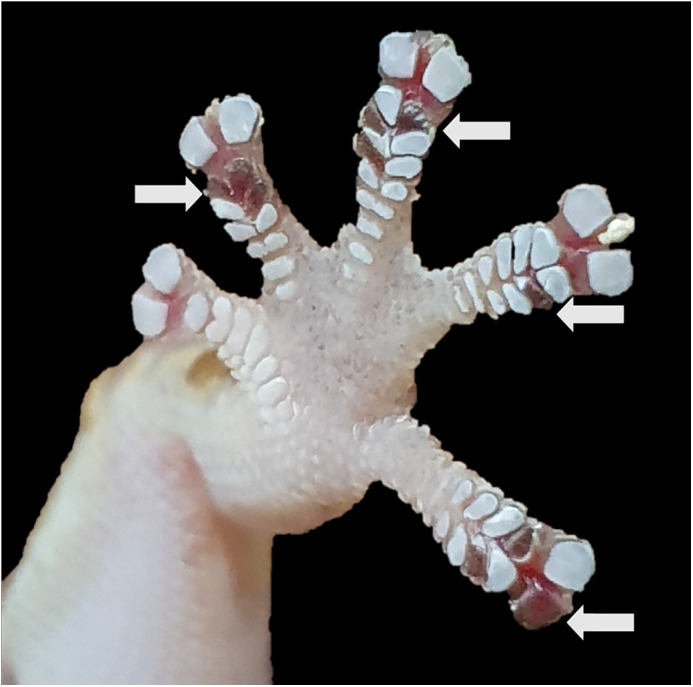
**Lamellae damage to gecko toepads on a macro-scale.** White arrows indicate regions where partial or entire lamellae have been separated from the likely mesos layer (photo taken with a Samsung^®^ Galaxy S10 at 2× magnification).

Macroscopic damage to toepads was obvious in 11/29 geckos during experiment 1. Toepads were entirely removed, leaving parts of the mesos layer exposed. No such damage was ever observed on geckos within 5 days of shedding. Damage on the micro- and nano-scale may have occurred, but could not be observed without destructive microscopy (Fig. 3).

### General use and ecdysis (experiment 2)

The change in shear force before versus after shedding, when no ‘extra’ damage was induced, was best predicted (lowest AICc) by a model including an interaction among species, treatment and days before shedding, which also included toepad area and mass as scaled, fixed terms (*R*^2^ conditional=0.89). The significant three-way interaction indicated that the slopes of the change in shear force before and after shedding were different among species (or that the amount of improvement induced by ecdysis varied among species), and also varied with the number of days before shedding the measurement was taken. The interaction among species, treatment and days before shedding (*P*<0.01, χ^2^ =34.60, ANOVA, Type III Wald χ^2^ tests), mass (*P*<0.01, χ^2^=4.3, ANOVA, Type III Wald χ^2^ tests) and toepad area (*P*=0.06, χ^2^=3.59, linear mixed-effects model, ANOVA, Type III Wald χ^2^ tests) were significant variables in the best model (Table 2). Maximum shear force was significantly higher 5 days following shedding compared with just before it in most species; however, the magnitude of increases in clinging ability varied among species

**Fig. 4.**
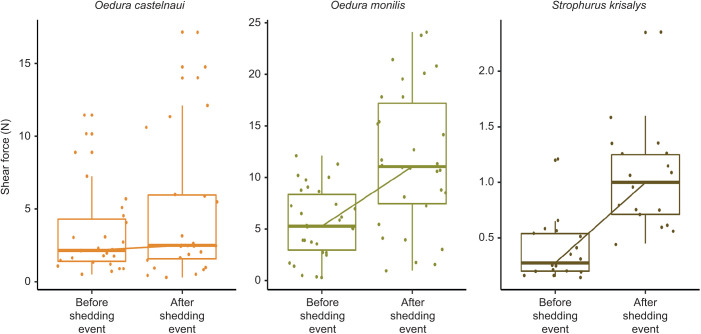
**The role of ecdysis in restoring clinging ability in three gecko species after general use.** Absolute shear force (median±s.e.) before and after a shedding event. Following ecdysis, clinging ability increased by 5.23 N in *O. monilis* (*N*=11, *P*<0.01, estimated marginal least square means *post hoc* comparisons), and by 5.67 N in *S. krisalys* (*N*=6, *P*<0.01, estimated marginal least square means *post hoc* comparisons). Clinging ability increased in *O. castelnaui* (*N*=9); however, this increase was not statistically significant. Note that *y*-axis scales have been adjusted to reflect differences in magnitude of clinging ability exerted by each species. Color palette using R package colRoz.

Generally, with no extra induced damage, mean clinging ability still increased following shedding in all species, and the increase was statistically significant in *O. monilis* (*N*=11, *P*<0.01, estimated marginal least square means *post hoc* comparisons) and *S. krisalys* (*N*=7, *P* <0.01, estimated marginal least square means *post hoc* comparisons). The increase in clinging ability following ecdysis was not statistically significant in *O. castelnaui* (*N*=9, *P*=0.3, estimated marginal least square means *post hoc* comparisons). Following ecdysis, clinging ability increased by 5.23 N in *O. monilis* and by 5.67 N in *S. krisalys* (Fig. 4). The magnitude of increase in clinging ability were stronger in some species than others and depended on the number of days since shedding (Fig. S2).

### Clinging ability after damage and after general use

**Fig. 5.**
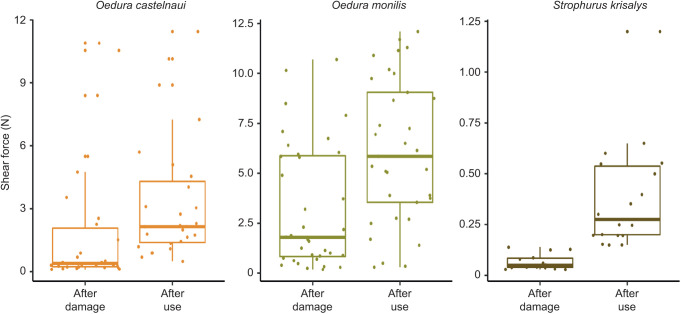
**Clinging ability in three gecko species after damage and after general use.** Clinging ability (median±s.e.) after induction of damage (30 days after a shedding event). Decline caused by damage was 1.52 N greater than that caused by general use in *O. castelnaui* (*N*=10 in experiment 1; *N*=9 in experiment 2; *P*<0.01), 2.80 N in *O. monilis* (*N*=11, *P*<0.01) and 0.26 N in *S. krisalys* (*N*=6, *P*<0.01).

Damage caused a greater reduction in shear force compared with general use. The interaction between ‘species’ and ‘test period’ significantly affected the shear force exerted after damage and with general use (*P*<0.05, χ^2^=6.88, linear mixed-effects model, ANOVA, Type III Wald χ^2^ tests). Decline caused by damage was 1.52 N greater than that cause by general use in *O. castelnaui* (*P*<0.01), 2.80 N in *O. monilis* (*P*<0.01) and 0.26 N in *S. krisalys* (*P*<0.01, Fig. 5).

### Safety factors

**Fig. 6.**
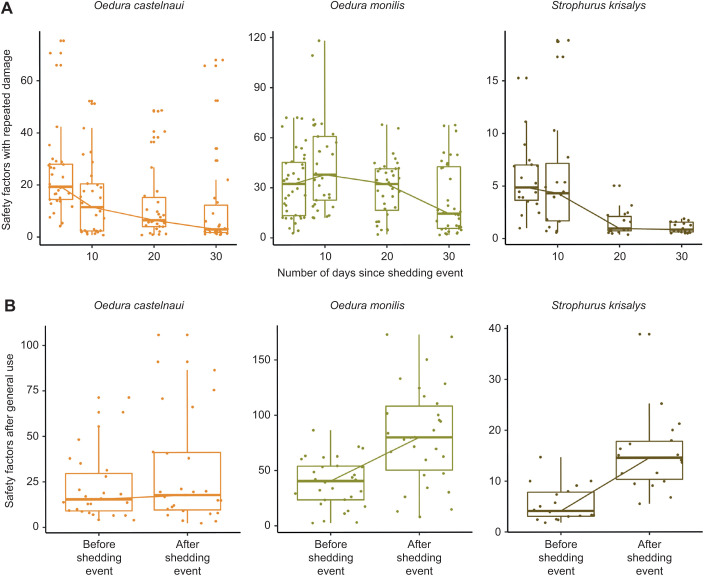
**Safety factors in three gecko species after damage and after general use.** (A) Safety factors (median±s.e.) associated with absolute shear force in relation to repeated damage. *O. castlenaui* (*N*=10), *O. monilis* (*N*=11) and *S. krisaly*s (*N*=6). (B) Safety factors (±s.e.) associated with absolute shear force with general use and after ecdysis. *O. castlenaui* (*N*=10), *O. monilis* (*N*=11) and *S. krisalys* (*N*=6). Color palette using R package colRoz.

The best model (lowest AICc) predicting the differences in safety factors in relation to increasing mechanical damage (experiment 1) included the interaction between ‘species’ and ‘days since shedding’ (*R*^2^ conditional=0.61, linear mixed-effects model, ANOVA, Type III Wald χ^2^ tests) consistent with the best model predicting the influence of damage on clinging ability (above). Safety factors decreased significantly in *O. castelnaui* between day 5 and day 30 (*P*<0.05), and between day 10 and day 30 in *O. monilis* (*P*<0.01). The decrease in safety factors between day 5 and day 30 was not statistically significant in *S. krisalys* (*P*=1). We concluded that the slope of the decline in safety factors with damage differed among species because the best model predicting the decline in safety factors with increasing damage included an interaction between ‘species’ and ‘days since shedding event’. Safety factors reduced by 12.54 in *O. castlenaui* between day 5 and day 30 after damage, 9.87 in *O. monilis* between day 10 and day 30 after damage. Safety factors decreased by 4.77 in *S. krisalys*, but this decline was not statistically significant (Fig. 6A).

The best model predicting the change in safety factors before and after ecdysis (experiment 2) included the interaction between ‘species’ and ‘days since shedding’ (*P*<0.01, χ^2^ =27.36, ANOVA, Type III Wald χ^2^ tests) and ‘species’ by itself (*P*<0.01, χ^2^ =24.95, ANOVA, Type III Wald χ^2^ tests). Safety factors were significantly lower by 36.54 prior to ecdysis in *O. monilis* (*P*<0.01) and by 9.27 *S. krisalys* (*P*<0.01); however, they did not change significantly in *O. castelnaui* (*P*=0.84, Fig. 6B).

## DISCUSSION

We show that macro-scale damage in the form of ruptured scansors or lamellae, most likely combined with additional damage on the micro- and nano-scale, lead to a decline in clinging ability in all three species we examined, with an associated reduction in safety factors. After ecdysis, clinging ability increased in all three species, although not significantly in *O. castelnaui*. Even without exposure to ‘extra’ damage at consistent intervals, shear force was lower prior to ecdysis. Possibly, just walking and contacting substrates causes mechanical damage reducing shear force. Alternatively, clumping or fouling of setae may be occurring (Hiller, 1968), or perhaps all three mechanisms reduce the effectiveness of setae over time. Thus, we conclude that both damage and general use affected attachment capabilities in these geckos. It was clear from our studies that the periodic process of ecdysis restored attachment capabilities and significantly increased safety factors.

### Damage and clinging ability (experiment 1)

While some early studies suggested that declines in performance with time since shedding may be caused by damage and clumping of setae caused by accumulation of contaminants (Hiller, 1968), other studies have found little evidence for declines in performance at the level of individual setae (Gravish et al., 2010; Puthoff et al., 2013; Autumn et al., 2014). In our study, especially when we induced damage, we attribute the majority of the decline in performance to physical damage on the macro-scale, possibly with some breakage of individual setae or entire fields (nano- and micro-scale, respectively). When we did not induce damage, the reduction in clinging ability is difficult to explain. There may be damage at the micro-scale or clumping and fouling of the setae may have occurred. Evidence exists that setae have self-cleaning mechanisms (Hansen and Autumn, 2005), and because our geckos were housed on paper towel in plastic enclosures, the opportunity for microdamage and fouling seem low, but we cannot dismiss them as factors causing the decline. More detailed studies of the role of natural micro-scale damage and fouling are required, to clarify their roles in loss of clinging ability over time, especially for geckos using natural substrates.

There are six stages in the shedding cycle of geckos, which comprise a very long stage 1, followed by a rapid series of shorter stages just before shedding (Maderson, 1966). Depending on the stage of shedding, attachment could vary. In our study, the declines in shear force we quantified very likely occurred entirely in stage 1, the resting phase. We observed no consistent patterns of decline in shear force in relation to shedding phases, for example, there were no sudden drops late in testing as other shedding phases may have been reached. Future studies should investigate clinging ability, without the effect of damage, in the different shedding phases, to understand possible influences of the shedding cycle on clinging ability.

With damage, decreasing shear force caused reductions in safety factors, the extent of which was different among species. In our study, safety factors declined in *O. castelnaui* (24.42 to 11.88) and *O. monilis* (31.98 to 22.11) with increasing mechanical damage. Safety factors were consistently low in *S. krisalys*, which reflects the low shear forces throughout the entire experiment, and dropped close to 1 (5.77 to 1.03). Safety factors below 1 indicate failure of the adhesion system (Higham et al., 2017); therefore, repeated damage influenced adhesive capacity to a level close to where geckos could not support their body weight.

### Use and ecdysis (experiment 2)

Following experiment 1, geckos were allowed to shed before being tested for experiment 2. This gave us a measure of the renewal provided by ecdysis, without beginning from a (possibly) artificially low point after extensive damage. The opportunity for damage due to strong attachment was minimal in the second experiment; however, the attachment system was subjected to interactions with substrates within the enclosure. Although there was no induced damage, and potentially minimal other factors reducing clinging ability, which we could not quantify in our study, clinging ability was still lower before, and increased after ecdysis in this experiment. Hsu et al. (2012) found ‘gecko footprints’ of phospholipid residue left behind on surfaces close to a shedding event. They suggested that these lipid molecules may be a sacrificial layer, that protects the degradation of the β-keratin spatulae. Furthermore, the quantity of these lipids on setae is likely to affect the ductility of these structures. Therefore, the concentration of these lipids in setae prior to and after ecdysis might cause the differences in clinging ability we observed before and after a shedding event. Furthermore, the declines in clinging ability due to ‘use only’ were lower than the declines caused by ‘damage’ in all three species (Fig. 5).

We could not predict exactly when geckos would shed and as we were avoiding repeated testing, the number of days before ecdysis varied in experiment 2. Interestingly, our geckos' ability to attach varied in relation to the time of testing: there was a greater decrease in clinging ability of geckos as they approached ecdysis. If damage, clumping or fouling (or likely all three) accumulate over time, for those individuals tested immediately before ecdysis (as little as 1 day before), clinging ability should have been closer to its lowest value, whereas those tested earlier (as many as 43 days before) had higher clinging ability. Thus, the time of testing in relation to ecdysis influenced measures of attachment, suggesting that performance declines close to shedding, consistent with our first experiment. Another possibility is that the process of ecdysis itself lowers performance, perhaps by increasing the likelihood or severity of damage late in the cycle (Watson, 1971) or by reducing clinging ability by directly reducing setal performance. This kind of effect may be similar to other negative effects of shedding, such as clouded eyes in snakes (Brown, 1956) and soft shells in crabs (Watson, 1971) before ecdysis. It would be interesting to determine if use of certain orientations or angles of attachment, boldness or other behaviors requiring good clinging performance change as ecdysis approaches in geckos.

In experiment 2, when the attachment system was exposed only to substrate interactions through locomotion, safety factors did not increase significantly after ecdysis in *O. castelnaui*; however, they increased following ecdysis in *O. monilis* and *S. krisalys*. Clinging ability before and after ecdysis was highly variable (three out of nine individuals exhibited lower clinging ability following ecdysis) in *O. castelnaui*, potentially because of the differences in the level of interactions with substrates upon which they moved. Our findings indicate that even in a scenario where damage was not excessive, ecdysis did improve clinging ability and associated safety factors in *O. monilis* and *S. krisalys*. Even though safety factors were lower prior to ecdysis, they were still greater than 1, indicating that animals could still support their body weight despite declining performance over time. However, when damage was induced (experiment 1), safety factors fell to critically low levels in *S. krisalys*, which exhibited lower initial safety factors. This effect of damage indicates the attachment system's capacity to compensate for abrasion from general use if damage is not extensive; however, in instances when damage is extensive, safety factors can be lowered to a point where geckos cannot support their body weight using setae, which may indicate a role for claws.

Attachment mechanisms and their underlying morphology have been the focus of several studies over the past two decades (Federle, 2006; Niederegger and Gorb, 2006; Van Casteren and Codd, 2010; Bennemann et al., 2011; Hagey et al., 2014). Other studies have investigated differences in performance in relation to varying substrate characteristics (Langowski et al., 2019; O'Donnell and Deban, 2020; Stark and Yanoviak, 2020; Palecek et al., 2021; Song et al., 2021). Interactions with these substrates are likely to cause wear, damage or contamination, influencing performance (Hiller, 1968; Dellit, 1934). Here, we found that induced damage and general substrate interaction reduce performance in geckos, which is restored through ecdysis. Other attachment systems, for example, in cockroaches, spiders and stick insects are likely to undergo damage or wear as well, but the effect of damage on these attachment systems has received little attention. Self-cleaning can restore attachment in dock beetles (*Gastrophysa viridula*) and stick insects (*Carausius morosus*) (Clemente et al., 2010); however, to our knowledge, improvement in performance after use and damage has rarely been investigated. The role of ecdysis to repair damage in general, such as lost limbs or open wounds are well studied in invertebrates (Lai-Fook, 1968; Weis, 1976; Vafopoulou, 2009; Pellett and O'Brian, 2019), as are the underlying endocrine processes that enable ecdysis (Park et al., 2002; Žitňan et al., 2003; Zhu et al., 2019) but further studies could investigate the effect of ecdysis on performance in other attachment systems.

Our study highlights the influence of interactions with the environment on adaptations of epidermal outgrowths that play important functional roles, specifically for movement. Furthermore, we show how an obligate physiological process, ecdysis, plays a role in the repair and rejuvenation of morphology critical to movement. Differences in morphology and structure of naturally used substrates likely influence how prone setal fields are to damage. Hence, future studies should investigate damage and use in relation to morphological differences, and structure of substrates or terrain on which movement occurs.

## Supplementary Material

10.1242/jexbio.245286_sup1 Supplementary information
