# Supplementary material for: The role of ecdysis in repair of an attachment system: a case study using geckos
Source: J Exp Biol. 2023 May 12;226(10):jeb245286. doi: 10.1242/jeb.245286 (PMC10184769; doi:10.1242/jeb.245286)
Supplement: Supplementary information [file jexbio-226-245286-s1.pdf]

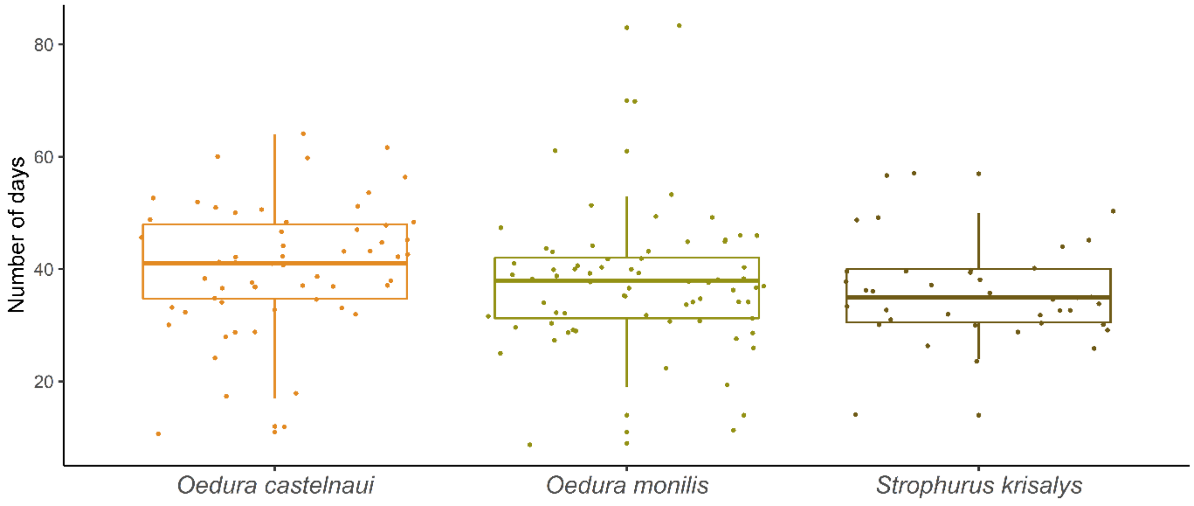

Fig. S1. Shedding intervals of three species of geckos. *Oedura castlenaui* (N=10), *O. monilis* (N=11) and *Strophurus krisalys* (N=7). Shedding intervals did not vary among species. Colour palette using r-package colRoz, Kong J, Wu N (2019). _colRoz: A colour palette for the land down under_. R package version 0.2.2, https://jacintak.github.io/project/colRoz

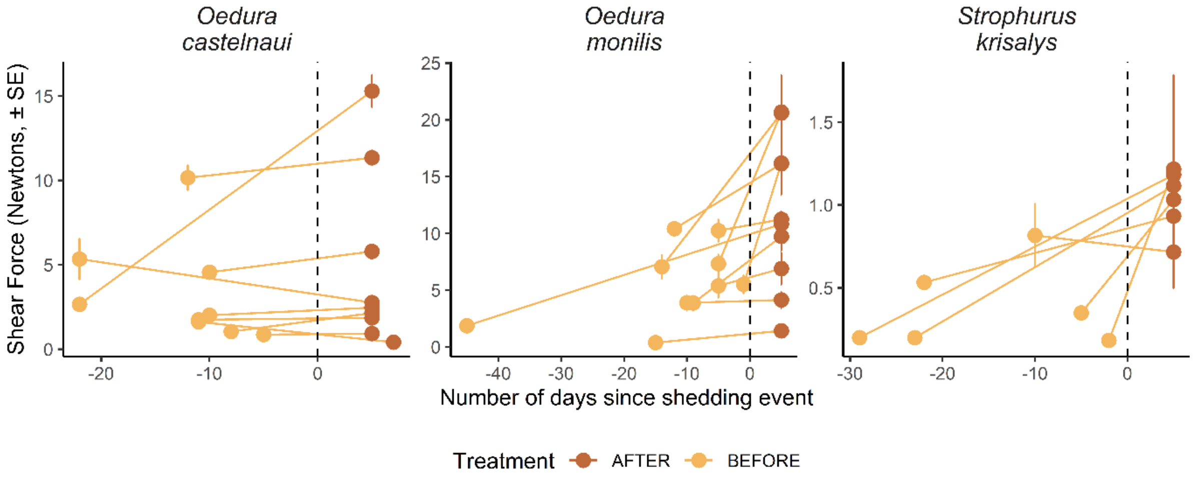

Fig. S2. Shear force after ecdysis in individuals. Absolute shear force (Newtons, 1-43 days) before a shedding event, and five days after shedding, from the top model (lowest AICc) that included the interaction between number of days before shedding, when maximum shear force was measured, species, mass and toepad area. Note: Y axis scales have been adjusted to reflect differences in magnitude of clinging ability exerted by each species. *Oedura castlenaui* (N=9), *O. monilis* (N=11) and *Strophurus krisalys* (N=7). Colour palette using r-package colRoz, Kong J, Wu N (2019). _colRoz: A colour palette for the land down under_. R package version 0.2.2, https://jacintak.github.io/project/colRoz
